# Thermo-Mechanical Behaviour of Human Nasal Cartilage

**DOI:** 10.3390/polym12010177

**Published:** 2020-01-09

**Authors:** Aureliano Fertuzinhos, Marta A. Teixeira, Miguel Goncalves Ferreira, Rui Fernandes, Rossana Correia, Ana Rita Malheiro, Paulo Flores, Andrea Zille, Nuno Dourado

**Affiliations:** 1 CMEMS-UMinho, Departamento de Engenharia Mecânica, Universidade do Minho, Campus de Azurém, 4804-533 Guimarães, Portugal; afertuzinhos@dem.uminho.pt (A.F.); pflores@dem.uminho.pt (P.F.); 2 2C2T—Centro de Ciência e Tecnologia Têxtil, Universidade do Minho, Campus de Azurém, 4804-533 Guimarães, Portugal; martaalbertinateixeira@gmail.com (M.A.T.); azille@2c2t.uminho.pt (A.Z.); 3 Department of Otolaryngology, Head and Neck Surgery, Santo António Hospital, 4099-001 Porto, Portugal; mgferreira.md@gmail.com; 4 HEMS—Histology and Electron Microscopy, i3S-Instituto de Investigação e Inovação em Saúde, University of Porto, 4200-135 Porto, Portugal; rfernand@ibmc.up.pt (R.F.); rcorreia@ipatimup.pt (R.C.); ana.malheiro@ibmc.up.pt (A.R.M.); 5 IBMC—Instituto de Biologia Molecular e Celular, University of Porto, 4200-135 Porto, Portugal; 6 Ipatimup—Institute of Molecular Pathology and Immunology of the University of Porto, 4200-135 Porto, Portugal

**Keywords:** cartilage, thermo-mechanical characterization, viscoelasticity, nasal soft tissue, rhinoplasty

## Abstract

The aim of this study was to undergo a comprehensive analysis of the thermo-mechanical properties of nasal cartilages for the future design of a composite polymeric material to be used in human nose reconstruction surgery. A thermal and dynamic mechanical analysis (DMA) in tension and compression modes within the ranges 1 to 20 Hz and 30 °C to 250 °C was performed on human nasal cartilage. Differential scanning calorimetry (DSC), as well as characterization of the nasal septum (NS), upper lateral cartilages (ULC), and lower lateral cartilages (LLC) reveals the different nature of the binding water inside the studied specimens. Three peaks at 60–80 °C, 100–130 °C, and 200 °C were attributed to melting of the crystalline region of collagen matrix, water evaporation, and the strongly bound non-interstitial water in the cartilage and composite specimens, respectively. Thermogravimetric analysis (TGA) showed that the degradation of cartilage, composite, and subcutaneous tissue of the NS, ULC, and LLC take place in three thermal events (~37 °C, ~189 °C, and ~290 °C) showing that cartilage releases more water and more rapidly than the subcutaneous tissue. The water content of nasal cartilage was estimated to be 42 wt %. The results of the DMA analyses demonstrated that tensile mode is ruled by flow-independent behaviour produced by the time-dependent deformability of the solid cartilage matrix that is strongly frequency-dependent, showing an unstable crystalline region between 80–180 °C, an amorphous region at around 120 °C, and a clear glass transition point at 200 °C (780 kJ/mol). Instead, the unconfined compressive mode is clearly ruled by a flow-dependent process caused by the frictional force of the interstitial fluid that flows within the cartilage matrix resulting in higher stiffness (from 12 MPa at 1 Hz to 16 MPa at 20 Hz in storage modulus). The outcomes of this study will support the development of an artificial material to mimic the thermo-mechanical behaviour of the natural cartilage of the human nose.

## 1. Introduction

**Figure 1:**
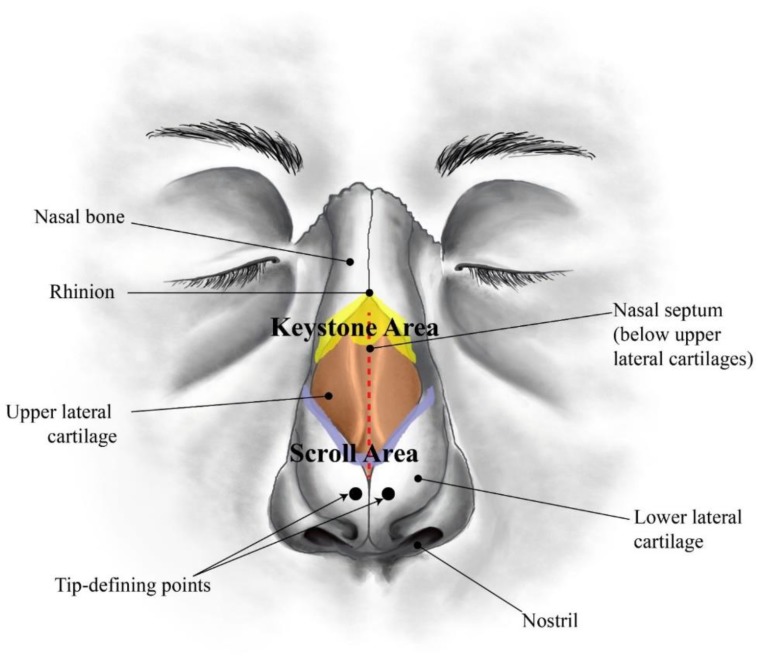
Sketch of a human nose showing the main anatomical regions (frontal view).

Soft tissues are among the most relevant biological structures with highly important mechanical functions. Tendons and ligaments, blood vessels, aortic valves, intervertebral discs, skin, and cartilage have been widely described in the literature from very early [1,2,3,4,5,6,7]. Cartilage is a resilient and viscoelastic tissue composed of specialized cells (chondrocytes) that is found in nose, ears, intervertebral discs, larynx, trachea, and thorax, as well as covering and protecting bones at the articular joints [8,9]. Due to its natural anisotropic organization, cartilage behaves differently in a variety of tensile and compressive modes [9,10,11,12]. Among these relevant cartilaginous structures, human nose has attracted numerous studies in the last few years related to surgical procedures [13,14,15]. The human nose is a very complex structure (Figure 1). The bony-cartilaginous pyramid of the external nose can be subdivided into: (i) an upper rigid portion composed by nasal bones (NB); (ii) a middle semi-rigid cartilaginous third composed by nasal septum (NS) and upper lateral cartilages (ULC); (iii) a lower flexible third combining the caudal edge of the NS with the lower lateral cartilages (LLC) [16,17]. Nose also comprises a set of soft neighbouring tissues around the bony-cartilaginous pyramid specifically within the well-known keystone (where NB variably overlap the ULC) and scroll (where ULC are overlapped by the cephalic edges of the LLC) areas [16,17,18,19,20]. These well-defined regions of transition are deeply studied because of their major relevance to nasal dorsum stability after any rhinoplasty act [16,21].

**Figure 2:**
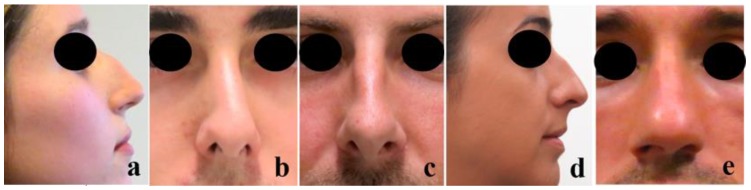
Most common nasal aesthetic defects: (**a**) Dorsal hump, (**b**) Crooked nose, (**c**) Wide dorsum, (**d**) Droopy nose (with hump), and (**e**) Rounded tip (the pictures were taken from the Miguel Ferreira’s patients by previous permission to be used in this study).

There are two main reasons to undergo a rhinoplasty intervention: functionality and aesthetics. The most common functional nasal disorder is nasal obstruction, mostly due to NS deviation, affecting approximately 80% of the worldwide population [22]. From an aesthetic point of view, the most common reasons for a rhinoplasty intervention are dorsal hump [23], crooked nose [24], wide dorsum [25], droopy nose [26], and rounded tip (Figure 2) [25].

Nowadays, the most performed surgical techniques to solve the aforementioned disorders are the septoplasty for correction of the NS deviation, osteotomies for nasal bones realignment, and humpectomies for dorsal hump reduction or removal [27,28]. Following hump removal, potential complications may occur due to loss of stability of the middle third, such as “inverted V” deformity, disruption of the dorsal aesthetic lines (DAL), or narrowing of the nasal dorsum caused by the ULC collapse, which creates insufficiency of the internal nasal valve [29,30].

The success of a rhinoplasty is fundamentally determined by the re-establishment of the normal airflow and dorsum appearance, which are a direct consequence of the mechanical behaviour of the involved biological materials. Nasal cartilages (NCs) are the most challenging structures to deal with due to their characteristic hyper-viscoelastic behaviour [31,32]. They belong to the group of hyaline cartilages that are mostly composed of chondrocytes and an extracellular matrix (ECM) rich in type II collagen and water (80%) [33,34,35]. NCs present superficial or peripheral, intermediate, and central zones [35,36]. Specifically, NS presents three zones of gradual transition in size, shape, and metabolic activity: (i) In the peripheral zone, there are small, flat, and oriented parallel cells to the cartilage surface, while (ii) the intermediate zone contains fewer, oval-shaped chondrocytes aligned perpendicular to the cartilage surface, and (iii) the central zone contains few, rounded chondrocytes arranged as single cells or aligned in columns perpendicular to the cartilage surface [35]. The ECM of the NS also shows differences in composition between peripheral and central zones. Previous histological staining studies have revealed the presence of a higher density of collagen fibres (type II) in the peripheral areas. Despite the fact that type I collagen and elastin were not present in NS, these can be found in the adjacent NS perichondrium [35]. Collagen fibres of ULC and LLC follow the curvature of the cartilage matrix, which can show high variability. Besides, many other solid constituents, such as aggregates of proteoglycans (PGs) composed of proteins and polysaccharides together with a small number of slightly rounded chondrocytes, are dispersed in the cross-linked fibrillar ECM [37].

Although water is the most abundant NC component, only a small portion is tightly bounded to the matrix. Thus, water within cartilaginous structures can be separated into fibrillar and intrafibrillar volumes [38]. Cartilage’s affinity for water is primarily justified by the presence of significant quantities of PGs, whose negative side chain charges attract free positive ions and increase internal osmotic pressure [39,40]. The remaining water is trapped within interfibrillar spaces of collagen or moving freely throughout the porous matrix, which contribute to the changes in mechanical properties of NC over time [39,41].

Although several studies have been performed on human lower limb joint and mandibular articular cartilages, very few works report on the characterization of the mechanical response of human nasal cartilages [42,43]. Despite sometimes well investigated beyond the physiological ranges of temperature and loading (between 37 and 100 °C and at 1 Hz on average), those studies were never performed in order to study the viscoelastic property of the material in absence of interstitial water. The evaluation of the viscoelastic properties of nasal cartilages at high temperatures and loading has never been reported in the literature, limiting the full comprehension of the material’s performance. Other studies, however, report on the development of polymeric composites as cartilage substitutes [44]. However, a comprehensive study on the thermo-mechanical properties of nasal cartilages aiming for the future design of a composite material to be used as artificial cartilage in the reconstruction surgery of the human nose is missing.

To fill this knowledge gap, in this work, the frequency-dependent viscoelastic properties of human NS, ULC, and LLC were studied using dynamic mechanical analysis (DMA) within the ranges 1 to 20 Hz and 30 to 250 °C. The output data were expressed in terms of loss (E’’) and storage (E’) moduli, as well as their ratio (tan∂). Further, general thermal features were also thoroughly characterized using thermogravimetry (TGA) and differential scanning calorimetry (DSC), in which weight loss (*WL*) and glass transitions were respectively determined, explained, and compared with benchmark literature. Variations of these parameters were assessed against frequency, anatomic location, and cartilage thickness, regarding their relative percentage of pure cartilage and soft adjacent tissues. The ultimate goal of this work is to provide a realistic, viscoelastic mechanical analysis under tension and compression loading of the human nasal cartilages. A comprehensive study of nasal structure behaviour may help to develop mechanically-matching biomaterial substitutes, as well as numerical prediction models.

## 2. Materials and Methods

### 2.1. Specimen Preparation

**Figure 3:**
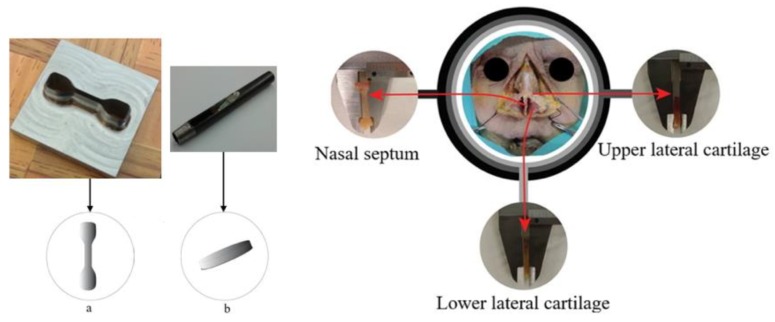
Harvesting of nasal cartilage samples in different regions of corpse nose: (**a**) *dog-bone*- and (**b**) flat disc-shaped specimens.

Nasal cartilage (NC) was harvested from two Caucasian male donors (Figure 3) through an endonasal cut, according to the established international ethical guidelines (complying with the Helsinki form) and in accordance with procedures approved by the Ethics Committee of CHUP/ICBAS of the University of Porto, Portugal (Project identification code: P2019-CE-P014). Through this procedure, the authors complied with institutional ethical use protocols. The cadavers were about the same age (70 and 82, respectively, donor 1 and 2), being well preserved. Periosteum and perichondrium layers were manually removed from NS, ULC, and LLC specimens (Figure 3). Remaining loose connective tissues were also carefully removed using a simple scissor to prevent slippage within the grips of the testing equipment, which is particularly relevant for the tension tests [45]. ‘Dog-bone’ and flat disc-shaped specimens were obtained using metal cutting matrices specifically designed and produced for this purpose. The geometrical configuration of the dog-bone specimens was established from the ASTM D412 standard, following proportional dimensions of the geometry reported in the referred standard (10 mm length and 2.5 mm width). The diameter of the flat discs was 7 mm.

Following a previous procedure, samples were carefully buffered with phosphate saline solution (PBS) to avoid dehydration during dissection and loss of tone of the tissues [46]. Specimens were then sealed in a plastic container and stored in a freezer at −40 °C to preserve the natural conditions of those tissues. Prior to mechanical testing, specimens were submitted to individual defrosting processes, which consisted in thawing the samples naturally at room temperature [47]. This preserving protocol (i.e., freeze-thaw treatment) has been previously shown not to change the dynamic mechanical properties of cartilage [43,48,49]. Further hydration of specimens was not executed, which corresponds to a quasi-dehydrated condition.

### 2.2. Specimen Measurements

Thickness and width were both accurately measured in different sections of each dog-bone specimen using a Vernier-calliper (resolution: 0.05 mm) and an open-source image processing software (ImageJ/Fiji to Java^®^ 8, Johannes Schindelin and coauthors, LOCI, University of Wisconsin, Madison, WI, USA). The resulting measures were obtained by calculating the mean value, drawing from ten different dimensions taken along the specimen thickness and width. This procedure allowed obtaining composite (cartilage and subcutaneous tissue) dimensions, as well as only cartilage. The average measurements of the cross-sectional areas of the specimens are summarized in Table 1. Thickness values of the flat disc-shaped specimen were also collected in diagonals, using the referred Vernier calliper from which the mean value was determined.

### 2.3. Dynamic Mechanical Analysis

Dynamic mechanical analysis (DMA) was performed using a DMA 7100 from Hitachi^®^ (Fukuoka, Japan) in programmed tension and compression methods. Specimens from NS were prepared in triplicate to conduct the mechanical analyses. These analyses were carried out in an atmosphere of nitrogen (200 mL·min^−1^) to ensure an inert environment.

#### 2.3.1. Tension Dynamic Analyses

The presented values for tension moduli were collected over a range of frequencies from 1 to 20 Hz in synthetic oscillation (the first frequency was set to 1 Hz, while the others were automatically attributed at 2, 4, 10, and 20 Hz). The temperature dependence of the tan∂, storage, and loss moduli (E’ and E’’) were measured in the temperature range of 30 to 250 °C at 3 °C min^−1^ (i.e., the heating rate). Dog-bone-shaped specimens, of approximately 10 mm length and 1.8 mm width, with 1 mm of thickness, and obtained with a stainless steel die cutting tool, were fixed to DMA metal grips without any glue or other interface material.

#### 2.3.2. Compression Dynamic Analyses

Values of compression moduli were collected in synthetic oscillation with frequencies of 1, 2, 4, 10, and 20 Hz. The temperature dependence of the tan∂, storage, and loss moduli (E’ and E’’) was measured in the temperature range of 30 to 250 °C at 3 °C min^−1^ (i.e., the heating rate). A disc-shaped specimen was produced with 7 mm diameter and approximately 1.3 mm height using a stainless steel punch tool.

### 2.4. Differential Scanning Calorimetry (DSC)

DSC measurements were performed in a Mettler Toledo DSC 822e apparatus (Mettler Toledo, Columbus, OH, USA). The analysis was carried out in nitrogen atmosphere (200 mL·min^−1^) to guarantee an inert environment. Specimens of cartilage (CT), composite (CP), and subcutaneous tissues (SC) harvested from NS, ULC, and LLC samples were submitted to a single heating step from 30 to 300 °C at a rate of 10 °C·min^−1^, being left to defrost until room temperature (23 °C) and placed in an aluminium pan. The initial mass was measured with a digital fully automatic Mettler Toledo calibration balance (AB 204-S/FACT Classic Plus, Mettler Toledo, Columbus, OH, USA). DSC curves were plotted with heat flow versus temperature. The main thermal events, melting temperature peaks, and enthalpies were determined.

### 2.5. Thermogravimetric Analysis (TGA)

Thermogravimetric analysis (TGA) was carried out in a STA 7200 Hitachi^®^ (Fukuoka, Japan), in which TGA and differential thermal analysis (DTA) are shown simultaneously. TGA plots were obtained within the range of 30–500 °C under nitrogen atmosphere (200 mL·min^−1^) at 3 °C·min^−1^. Specimens of cartilage (CT), composite (CP), and subcutaneous tissues (SC) harvested from NS, ULC, and LLC samples were left to defrost until room temperature (23 °C) and placed in an aluminium pan. The initial mass was measured (same equipment as in the previous section) prior to testing. Data was plotted as weight loss (*WL*) (in percentage) versus temperature, and the mass of dried residues calculated for each case. The derivative thermogravimetric (DTG) analysis was performed to identify the thermal transformation events (namely the maximum peaks).

### 2.6. Optical Microscope Analysis

Samples of soft tissues (NS, ULC, and LLC) were previously fixed in 10% buffered formalin and after 24 h off fixation were routinely processed in an automated system and embedded in paraffin. Sections were made at 4 µm in adhesive slides for H&E (Hematoxylin and Eosin).

### 2.7. Transmission Electron Microscopy (TEM) Analysis

Samples of soft tissues (NS, ULC, and LLC) were washed twice with PBS and fixed overnight with 2.5% glutaraldehyde/2% paraformaldehyde in cacodylate buffer 0.1 M (pH 7.4). Then, samples were washed in 0.1 M sodium cacodylate buffer and fixed in 2% osmium tetroxide in the 0.1 M sodium cacodylate buffer overnight, followed by a new fixation in 1% uranyl acetate overnight. Dehydration was performed in gradient series of ethanol solutions and propylene oxide and included in Epon resin by the immersion of samples in an increasing series of propylene oxide to EPON (till 0:1 ratio) for 60 min each. Sample inclusion in EPON resin was performed in a silicon mould. Semi-thin sections with 500 nm thickness were prepared on a RMC Ultramicrotome (PowerTome, Labtech, Heathfield, East Sussex, UK) using a diamond knife and stained with Toluidine Blue. Electron micrographs were obtained using a Jeol JEM-1400 TEM (JEOL USA, Inc., Peabody, MA, USA) at 200 kV on naked copper grids coupled with a Orius SC 1100W digital camera (Orius, Pleasanton, CA, USA).

## 3. Results and Discussion

### 3.1. DSC Measurements

**Figure 4:**
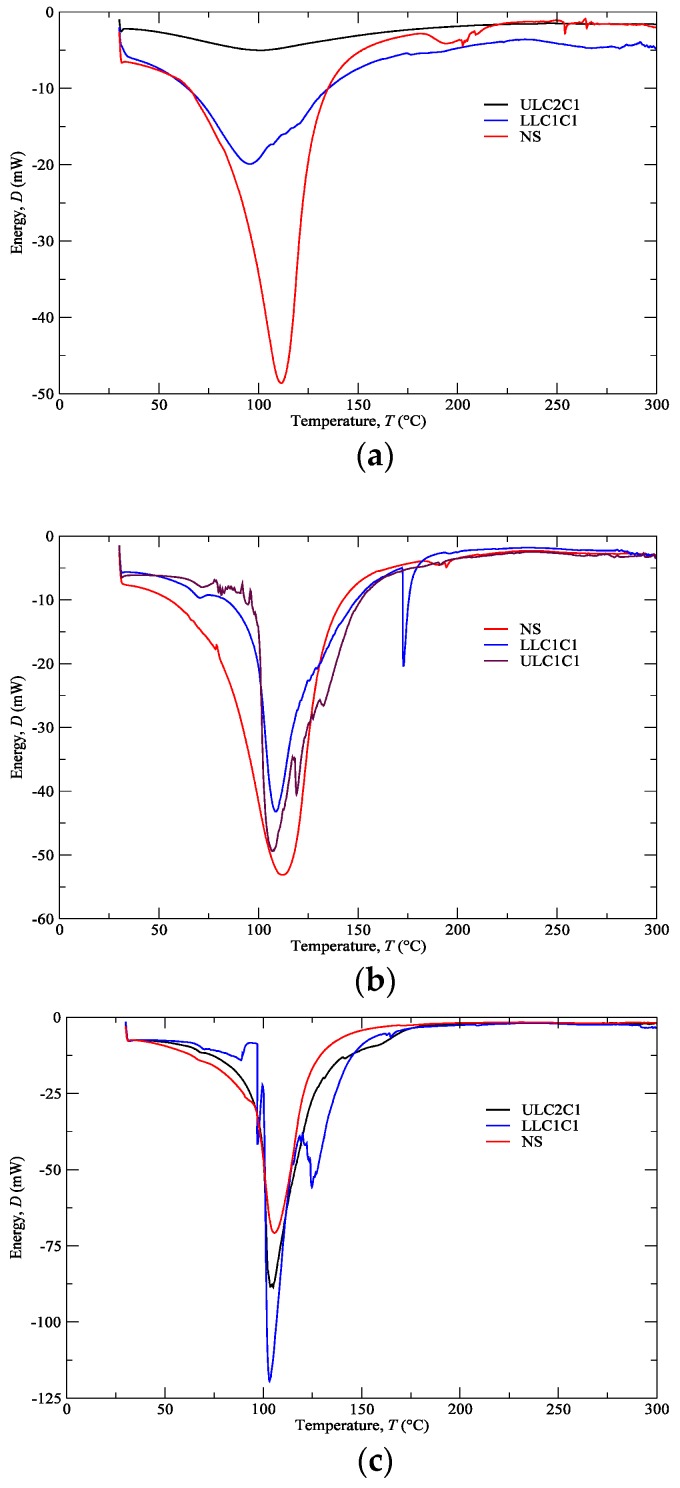
Differential scanning calorimetry (DSC) analyses of the (**a**) CT, (**b**) CP, (**c**) SC from NS, ULC, and LLC (donor 1).

**Figure 5:**
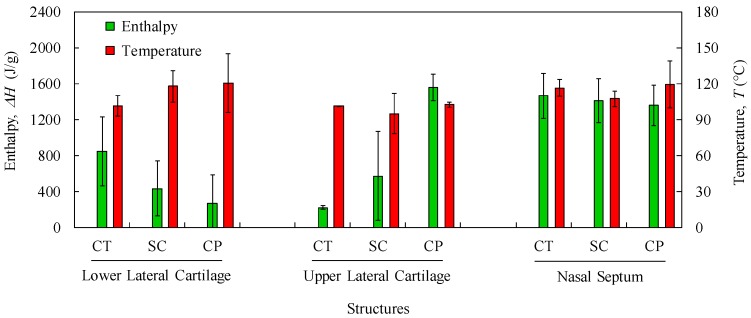
Average values and standard deviations of thermal features (Enthalpy and Temperature) of the 1st peak for LLC, ULC, and NS structures (CT: cartilage; SC: subcutaneous; CP: composite).

Figure 4 shows the plotting of energy dissipated with heat transfer within the interval of temperatures referred in Section 2.4 (i.e., 30–300 °C) for cartilage (CT), composite (CP), and subcutaneous (SC) tissues. These plotting data refer to nasal septum (NS), upper lateral cartilages (ULC), and lower lateral cartilages (LLC) of donor 1. Figure 5 resumes the main thermal features (enthalpy and temperature events) registered for the 1st peak.

Samples with higher changes in the enthalpy indicate that they require larger amounts of energy to undergo decomposition. A small glass transition *T*_g_ peak can be observed at 60–80 °C in the composite and subcutaneous tissue specimens (Figure 4b,c) due to melting of the crystalline region of collagen matrix. A main peak has been attributed to water evaporation between 100 and 130 °C for the total amount of specimens. However, no correlation among different nasal structures has been observed in the measured enthalpies (Figure 5) due to different water content. In general, a higher temperature peak (1st peak) can be depicted in the subcutaneous tissues compared to cartilage due to their high water binding capacity (also supported by TGA measurements).

Another interesting feature is the subcutaneous narrow water temperature peaks. On one hand, the cartilage and composite specimens show a wide large peak between 50 and 150 °C (Figure 4a,b). On the other hand, specimens of subcutaneous tissues display a smaller range of temperatures between 75 and 125 °C (Figure 4c). This result indicates once again the different nature of the binding water inside the studied specimens.

A second peak is also observed in the vicinity of 200 °C due to strongly bounded non-interstitial water in the cartilage and composite specimens (Figure 4a,b), but not in the subcutaneous tissues. No other significant thermal events can be observed up to 300 °C.

The obtained DSC results are important in terms of cartilage characterization since different enthalpies observed in nasal structures indicate that a potential substitutive material must be composed of distinct regions in terms of thermo-mechanical behaviour.

### 3.2. TGA Measurements

**Figure 6:**
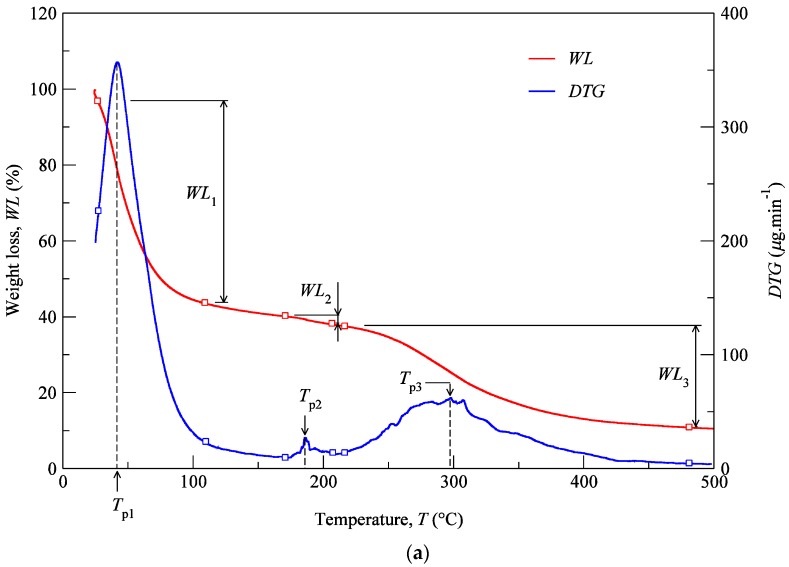

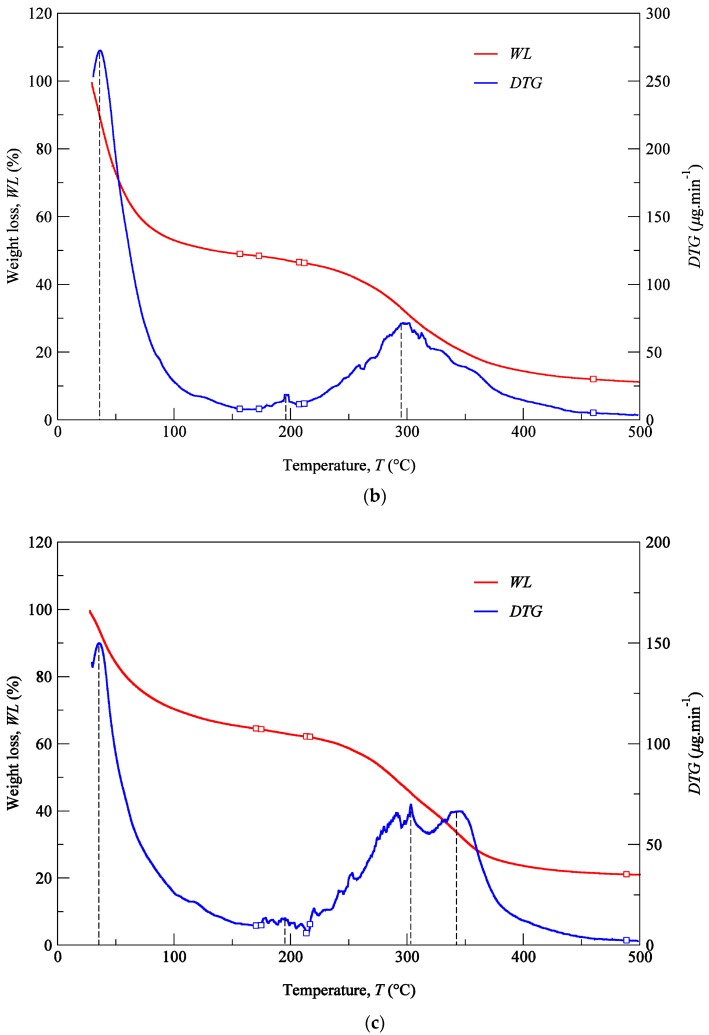
TG analyses of the LLC: (**a**) CT, (**b**) CP, (**c**) SC (donor 2).

**Figure 7:**
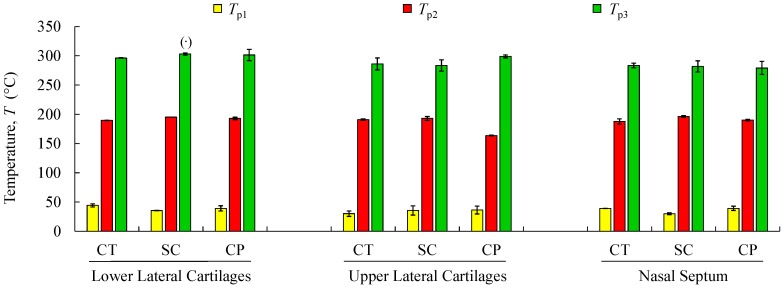
Mean values and standard deviations of thermal peaks (T_p1_ to T_p3_) obtained by TG/DTG analyses for the analysed structures regarding cartilage (CT), subcutaneous tissue (SC), and composite (CP). (**^·^**) Reports a single value.

**Figure 8:**
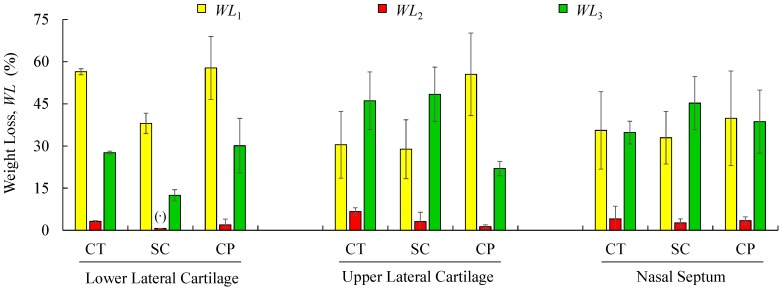
Mean values and standard deviations of weight-loss obtained by TG/DTG analyses for the analysed structures regarding cartilage (CT), subcutaneous tissue (SC), and composite (CP). (**^·^**) Reports a single value.

TGA was performed with the aim of identifying phase transitions (thermal degradations) in the material over time as the temperature changes. The used protocol (Section 2.5) imposed a heating rate of 3 °C min^−1^ on a set of samples constituted by cartilage (CT), composite (CP), and subcutaneous (SC) tissues. The results of the TGA are shown in Figure 6a–c for the specimens harvested from NS, ULC, and LLC samples of specimen 1, donor 2. In the referred figures, the *WL* curve represents the weight loss (in percentage) as a function of the temperature T (within the interval 30–500 °C), while the *DTG* curve stands for the first derivative of the TG over the same interval of temperatures. The first derivative peak, onset and endset temperatures (depicted pairs of small squares), corresponding weight losses (*WLi*), and mass of dried residues at 500 °C were determined for each case and registered in Figure 7 and Figure 8.

Thermogravimetric curves showed that the degradation of cartilage, composite, and subcutaneous tissue of the NS, ULC, and LLC take place in three well-defined thermal events (Figure 6), represented as *WLi* (Figure 7 and Figure 8). The three thermal events showed no significant differences among samples being divided in specific intervals of 30–44.2 °C for the first peak, 163.7–196.3 °C for the second peak, and 279.3–303.1 °C for the third peak.

Four peaks on the first derivative of the TG (see Figure 6a–c) were identified in LLC and ULC structures. The fourth peak was visible in the vicinity of 350 °C (namely the subcutaneous tissue from the LLC1 and the composite from the ULC1). This was attributed to contaminations of nose trimmers, fatty tissue, or bony/calcified cartilage traces. The residual weight at 500 °C that was measured has also been found very disperse, which does not allow for the establishment of a significant relation with a given biological structure.

Figure 7 shows mean values of the onset and endset temperatures corresponding to each thermal event registered for the biological structures. Hence, the first thermal event arises on average at 36.5 °C, which corresponds to the initial weight loss (in water). The weight loss due to water evaporation has been registered in 42% (on avg.), with high scatter (CoV: 33%), presenting values between 28.9 and 57.7 wt % (Figure 8). A second, small but consistent weight loss (overall average between 0.7 and 6.7 wt %, according to Figure 8) was observed at an average temperature of 188.8 °C, revealing that the weight loss in cartilage samples was always higher than in the remaining structures (on avg. 3.2 wt % for LLC, 6.7 wt % for ULC, and 4.1 wt % for NS, according to Figure 8). Above previous temperature interval, the weight continues to diminish until a third phase (on avg. at 290.5 °C) is revealed. A variable solid residue content at 500 °C between 8.2 and 19.5 wt % was observed for all the specimens. 

In contrast to other studies on hyaline cartilages that show high water content (up to 85%), in this work, cartilages have showed an average of 42 wt % water content [50]. It is reasonably plausible that a given donor can present different water content within the body parts due to natural dehydration. Another reason might be attributed to donors age, which could present high content in calcified cartilage, which is expected to have less water content.

The speed of the water loss represented by the first *DTG* peak and the weight loss (in percentage) reported in Figure 8 showed different behaviour in cartilages and subcutaneous tissues for LLC, ULC, and NS structures. It was found that cartilage releases more water and more rapidly than the subcutaneous tissues. A reasonable explanation for this behaviour may be attributed to proteoglycans and collagen molecules actions that promote a matrix with a very efficient water-binding capacity. However, water is predominantly weakly bounded to aminoglycan side chains of aggrecan molecules [51]. In general subcutaneous tissues, water content ranges between 6% and 26%, although there is no information in the literature regarding nasal subcutaneous tissues [52]. Subcutaneous tissues present a higher amount of adipose and collagen tissues. Collagen fibres produce a large surface area and form extensive networks with high water-binding capacity, supporting somehow the observed results [53]. The small peak detected in the range of 180–200 °C can be attributed to tightly bound water. In fact, chemically bounded water is estimated to be around 4% in cartilage, and it is expected to evaporate at temperatures around 200 °C [54]. The degradation that occurs on average at 290.5 °C (Figure 7) is attributed to the decomposition of protein structures, such as deamination, decarboxylation, and depolymerisation of the polypeptide bonds [55]. The solid residues at 500 °C seem to be in accordance with previous similar analyses [56].

The TGA analysis provides important information on the role of the interstitial water in cartilage structure integrity. Despite the fact that such temperatures will never be achieved in physiological conditions, the obtained data will be essential for the selection of materials to be used as cartilage substitutes.

### 3.3. Multi-Frequency Tensile and Compressive Loading of NS

DMA has been previously used to determine the viscoelastic properties of human lower limb joint and mandibular articular cartilages, with imposed frequencies from 0.1 to 10 Hz [42,43]. However, the evaluation of the viscoelastic properties of nasal cartilages at high temperatures and over a similar frequency range has never been studied. Typically, the literature analyses the relationship between oscillation frequencies and moduli at the physiological temperature of 37 °C or in a range of temperatures up to 100 °C. In this work, a relationship among frequencies (1–20 Hz), temperatures (up to 250 °C), and moduli was investigated to deeply understand the thermo-mechanical behaviour of nasal cartilages. 

**Figure 9:**
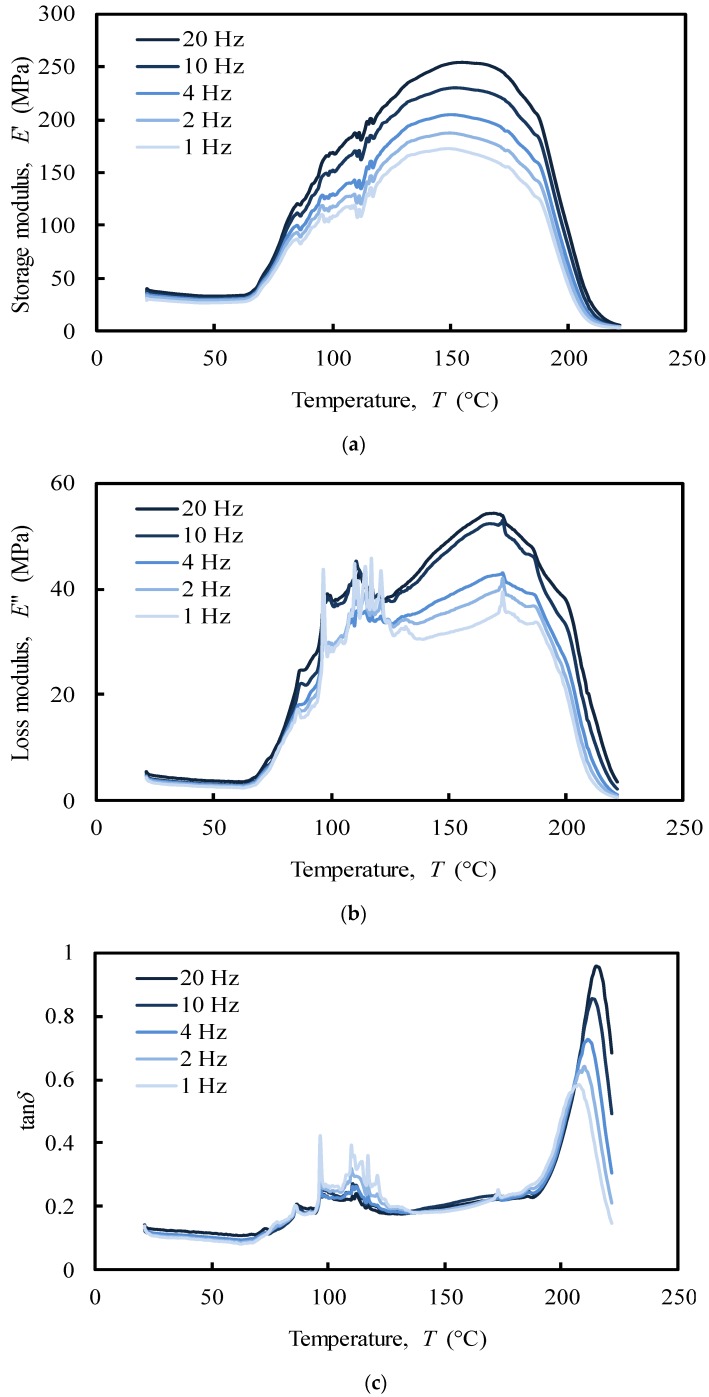
Tensile multi-frequency (**a**) storage and (**b**) loss moduli, (**c**) damping of the NS (donor 1).

**Figure 10:**
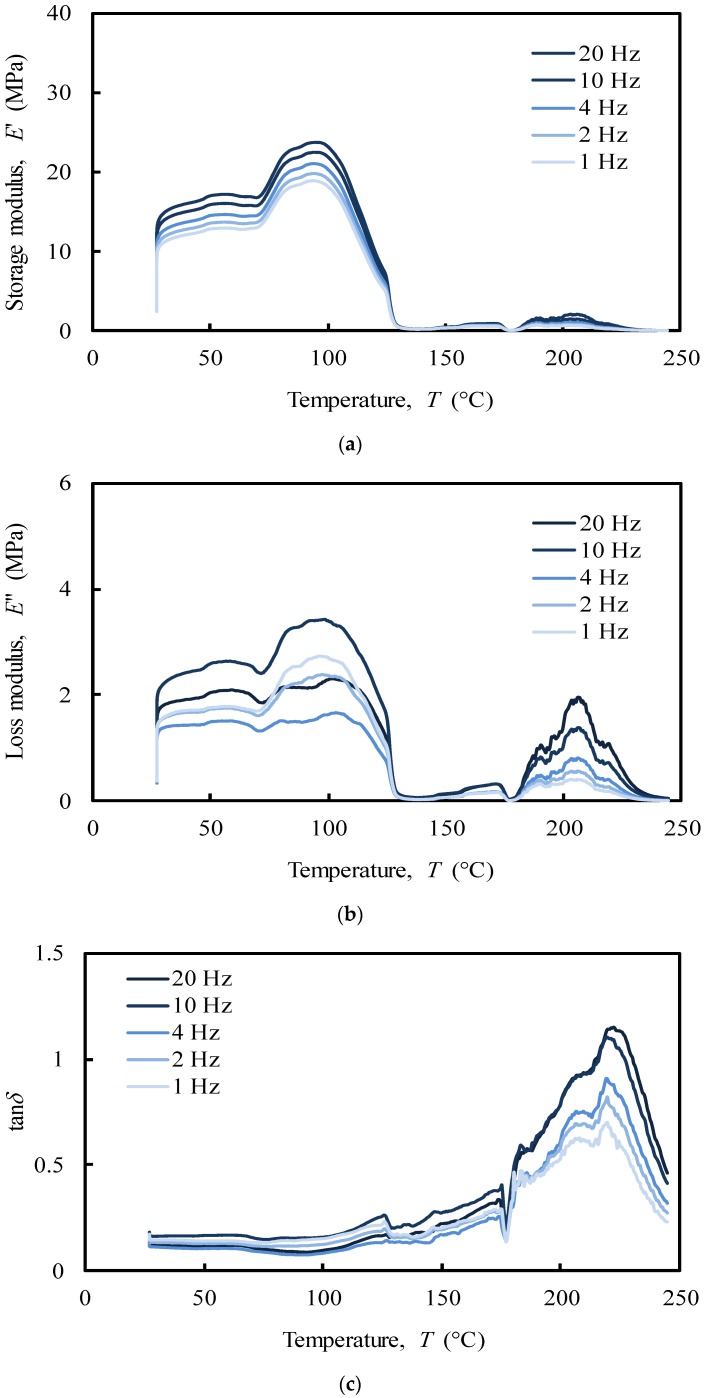
Compressive multi-frequency (**a**) storage and (**b**) loss moduli, (**c**) damping of the NS (donor 1).

The results of the DMA for tensile and compressive multi-frequency analyses are shown in Figure 9 and Figure 10, respectively, for cartilaginous tissues of the nasal septum (NS) that does not contain subcutaneous tissue (pure cartilage). As referred in Section 2.3.1, the results are expressed as storage (E’) and loss (E’’) moduli, and damping factor (tan∂). A high tan∂ value implies a material with a significant non-elastic strain component and a low value indicates one with a high elastic contribution [57].

The analysis of the tensile loading mode (Figure 9) shows a different behaviour of the NS structure comparatively to compressive loading mode (Figure 10). In the first case, a series of secondary frequency-independent peaks in the range of 70–100 °C (Figure 9c) was revealed due to melting of crystalline regions of collagen. The motions of the main and side chains of collagen molecules become significant in this region when a tensile force is applied, causing an irreversible spatial reorganization within the cartilage matrix [38,58]. This behaviour is in agreement with the different structural organization that collagen has been reported to be composed of: (*i*) an unstable crystalline region with little orientation that melts at low temperatures (80–180 °C), (*ii*) a natural amorphous region associated to second-order phase transitions that melts at around 120 °C, and (*iii*) a stable crystalline region with a melting point at 200 °C [59]. At the physiological temperature (app. 37 °C) no significant events in storage (E’) and loss (E’’) moduli were recorded (Figure 9a–b). This tissue is clearly frequency-independent in tensile mode at this temperature.

Regarding compression analysis, the system showed a peak in the storage modulus (E’) within the interval 90–100 °C (Figure 10a), which was noticed to increase with the imposed frequency (1–20 Hz). In the tensile analysis, the system showed a similar frequency-dependent behaviour, but with a maximum peak at around 150–160 °C in both storage and loss moduli (Figure 9a,b). Moreover, the referred moduli in tension are 10 times higher than the ones measured under compression. It has been previously shown that off-bone cartilage has a frequency-dependent loss modulus leading to an increase of energy dissipation at higher frequencies (e.g., 10 and 20 Hz) reducing the potential damage due to the increased hysteresis [49]. Nasal cartilage behaves very similarly in this case. However, it is worth remembering the importance of the internal swelling pressure of cartilage in compression. The collagen fibrils polyanionic matrix is able to endure a compressive stress maintaining a water swelling pressure by Donnan effect, which increase cartilage stiffness without damaging the collagen network [60].

The storage and loss moduli showed a maximum value of approximately 250 MPa and 50 MPa, respectively (Figure 9a,b). This can be attributed to the well-known high tensile modulus of collagen, though lower in compression [61].

In contrast to the compressive mode, the peak at approximately 200 °C in the tensile mode showed a frequency-dependent behaviour over the temperature in tan∂ curve (Figure 9c), which clearly indicates the presence of a glass transition point. The glass transition temperature (*T*_g_) of a polymer is a very important characteristic to understand the effects on the mechanical behaviour, the degree of crosslinking, and the changes that occur within the bulk at a specific temperature. Despite *T*_g_ measures are very common for synthetic polymers at high temperatures, little is known about the behaviour of protein polymers [62]. The increase in tan∂ peak temperatures with the imposed frequency (1–20 Hz) was used to calculate the apparent activation energy, by employing the frequency-temperature superposition principle (also known as time-temperature superimposition). Hence, an Arrhenius plot, in which the log of the imposed frequency is plotted against the inverse of the peak temperatures, was performed. The slope of the best-fit linearization of the obtained data corresponds to the apparent activation energy that was estimated as 780 kJ/mol. The value of the activation energy may provide important information about the degree of crosslink of viscoelastic polymers and on the nature of the molecular relaxation transitions. The observed activation energy is in the range of the previously observed thermal denaturation process of collagen matrices, indicating a relatively high physical entanglement of the collagen chains. However, the increase of tan∂ is frequently associated to material degradation, and to an increased mobility of the molecular chains that leads to increase the energy consumption [63]. Thus, the peaks at 200 °C suggest that the phase transition of dry collagen is more probably a denaturation than a melting process of the stable crystalline structure of collagen. The value of activation energy confirms that dry denaturation is a complex process, which probably consists in a rate-limited reversible step leading to an irreversibly denatured state of the proteins [64].

In the compressive analysis (Figure 10), at the physiological temperature (app. 37 °C), the storage modulus (E’) of the NS (Figure 10a) increases 33% with the imposed frequency (from app. 12 MPa at 1 Hz to app. 16 MPa at 20 Hz). This result is consistent with previous studies using similar frequency ranges [65,66].

The loss modulus (E’’) of the NS (Figure 10b) was, as expected, an order of magnitude lower than the storage modulus (Figure 10a). Regarding articular cartilage, in which restrictions caused by the bones prevent an increase in loss modulus with the imposed frequency, the characterization performed herein revealed that for nasal cartilages the loss modulus varies with the imposed frequency from approximately 1.4 MPa at 4 Hz to 2.4 MPa at 10 Hz, with an increasing rate of 71%. However, no linear relationship between oscillations and energy dissipated was found [49]. Indeed, previous studies have shown that the loss modulus of cartilage was insensitive to loading frequency under compression [65,66]. This behaviour can be correlated to the hydration degree of cartilages, which was observed to be frequency independent. It seems that the cartilage viscoelastic properties in unconfined compression are only triggered by the fluid mobility and structural interactions between reinforcing collagen and surrounding matrix gel [8,48,67,68,69,70].

The analysis of the tan∂ under compressive loading (Figure 10c), as a function of temperature up to 250 °C, exhibits three relaxation peaks with different elastic behaviour. These peaks, at around 120, 180, and 220 °C, are related to a second-order phase transition of the amorphous regions of collagen (app. 120 °C) and to melting of the crystalline zone of collagen (app. 200 °C) and proteoglycans (app. 220 °C) matrices, respectively [71,72]. However, since in the physiological state cartilage is heavily hydrated, these peaks are very likely to be associated to the mobility of water that is bounded to the cartilage solid structure. Despite the high loss of water, the peak at 120 °C shows tan∂ values ranging from 0.2 to 0.3, which indicates that under compressive loading the system is primarily elastic at this temperature, presenting a small, though not negligible viscosity. In fact, at 120 °C the system mostly adsorbs energy as it undergoes elastic deformation (high storage modulus), while little energy is dissipated as heat (low loss modulus) (Figure 10a,b). The peaks at 180 and 220 °C, fundamentally associated to the water strongly bonded to cartilage, are considerably higher denoting a predominant viscous behaviour. As tan∂ represents the measurement of the ratio of energy dissipated as heat to the maximum energy that is stored in the material, more energy is stored in the material at lower values of tan∂. At higher temperatures (app. 230 °C), a sharp drop of the tan∂ is observed due to the denaturation of proteins [73].

The observed differences between tensile and compressive modes can be attributed to different properties that control the viscoelastic cartilage performance. In this work, the unconfined compressive mode was clearly ruled by a flow-dependent behaviour caused by the frictional force of the interstitial fluid (fluid pressurization) that flows within the porous matrix. Besides the important collagen inherent viscoelasticity, tensile mode was instead ruled by a flow-independent process produced by the time-dependent deformability of the solid cartilage matrix that is strongly frequency-dependent, showing a clear glass transition point at around 200 °C [74,75]. These results will be important to define the deformation boundaries that the biomaterial should withstand and, once again, the importance of the water in the material structural behaviour.

### 3.4. Histology and TEM Analyses

**Figure 11:**
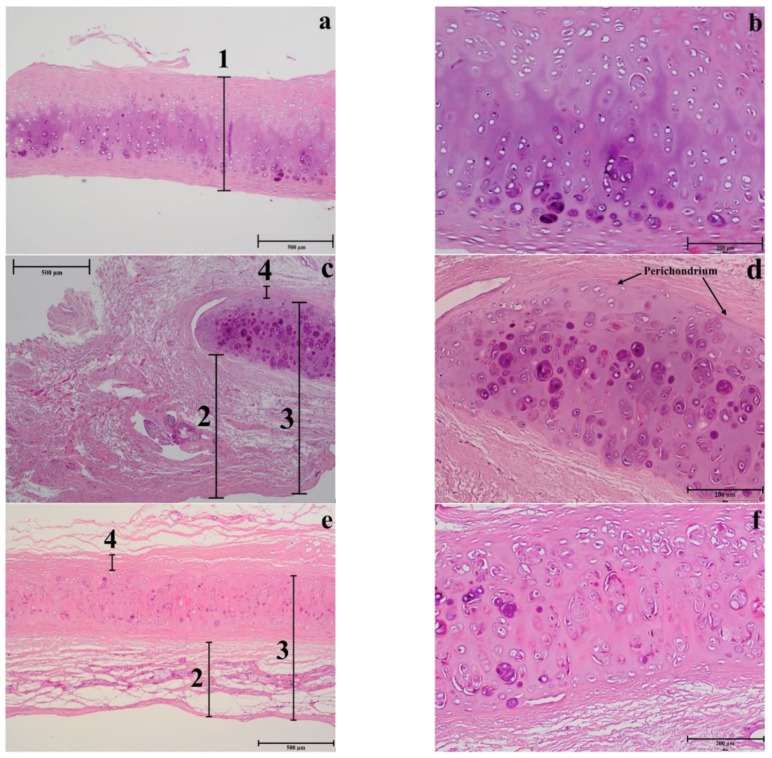
Histology of different nasal regions: of the NS (**a**–**b**), LLC (**c**–**d**), and ULC (**e**–**f**) samples. 1. Cartilage (µm); 2. Distance from the epidermis to the outer layer of cartilage (µm); 3. Distance from the epidermis to the inner layer of cartilage (µm); 4. Distance from the perichondrium to the tension area (µm).

Histological examination of NS, using Hematoxylin & Eosin stains, showed the presence of mature cartilage (Figure 11a,b). In both septal (Figure 11a,b) and upper (Figure 11e,f) cartilages, a clear transition between young and mature chondrocyte cells can be observed. Young cells are small and flat, situated near to peripheral zones where the matrix stains are pinkish, and oriented parallel to the cartilage surface, suggesting a rich collagen content. Mature cells are less in number with oval quasi-spherical shape and showed a 90-degree shift in cell orientation, showing a reddish colour specific of mature collagen [35].

**Figure 12:**
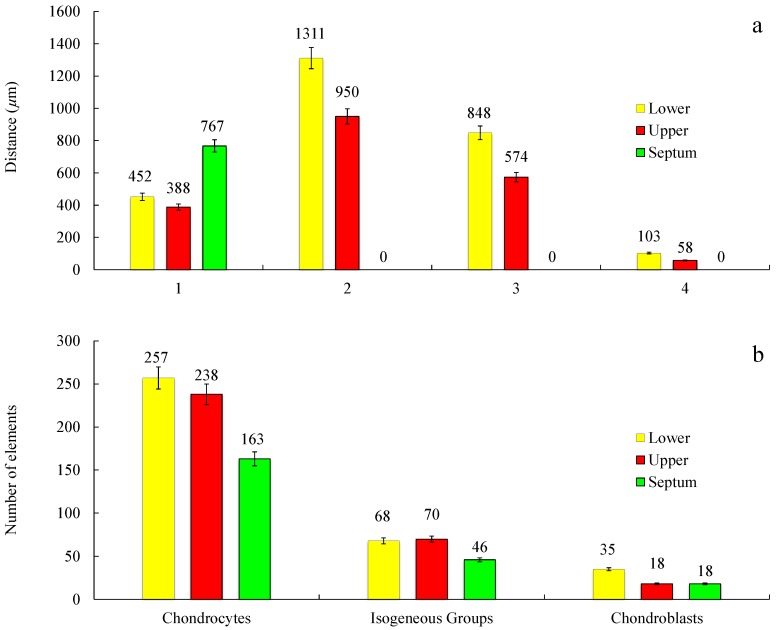
Measurements of different morphological regions (**a**) and count of different cell types (**b**) of the histology samples of the NS, LLC, and ULC. 1. Cartilage (µm); 2. Distance from the epidermis to the outer layer of cartilage (µm); 3. Distance from the epidermis to the inner layer of cartilage (µm); 4. Distance from the perichondrium to the tension area (µm). Cell count was evaluated in a sample area of 0.3641 mm^2^ (n = 3; SD < 5%).

In general, the results of nasal septum in relation to lower and upper regions presented a higher thickness and lower cellular content regarding all cell types, suggesting a greater constitution of hyaline type matrix, such as probably amorphous ground substance (glycosaminoglycans, proteoglycans, structural glycoproteins) and fibrils (elastic and type II collagen) (Figure 12b).

In the septum, the mature cells are concentrated in one side of the intermediate zone leaving an ECM central zone with a very low cellular density (Figure 11b). Differently, the upper cartilage is more symmetric showing an even cell distribution (Figure 11f). Moreover, septal cartilage is thicker (Figure 12a) and more rigid than upper and lower ones and did not show any tension area after the perichondrium, because of its function of providing rigidity to the human nose. Upper and lower cartilages displayed morphologically different and well-defined tension areas due to their cartilage functional differences.

**Figure 13:**
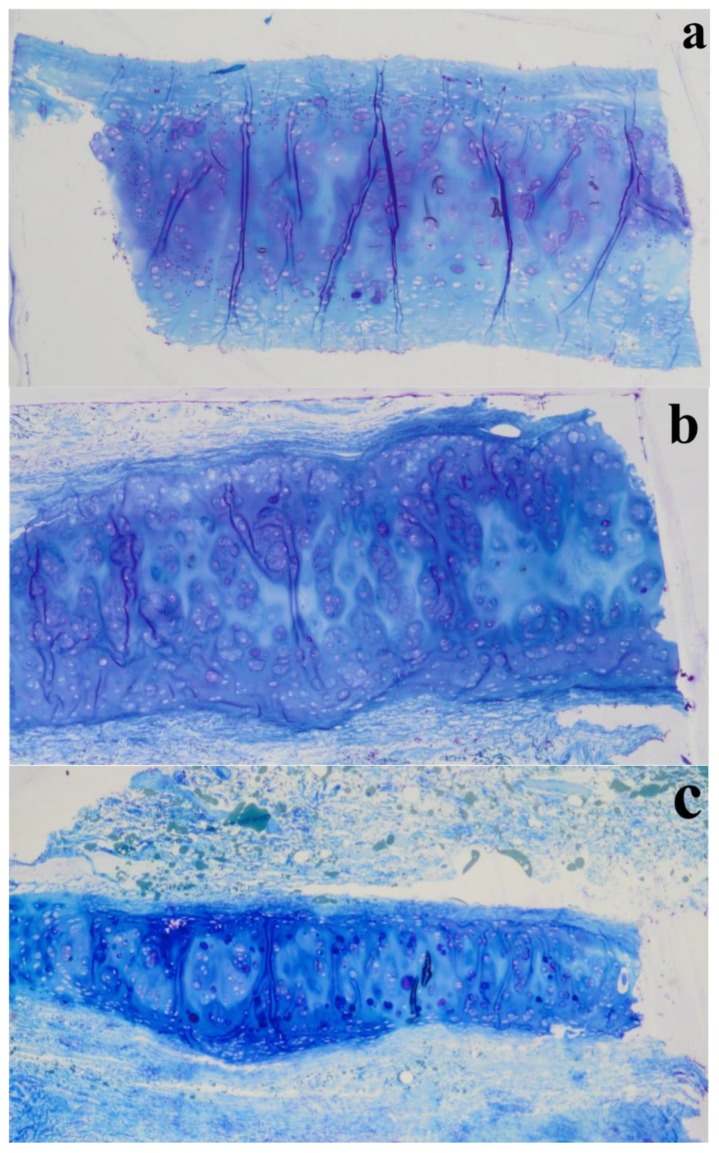
Semi-thin sections (1 µm thickness) stained with Toluidine Blue of different nasal regions: (**a**) NS, (**b**) LLC, and (**c**) ULC.

As previously observed, differently to ear cartilage, the nasal perichondrium is strongly attached to the cartilage without a well-defined transition zone [76]. However, the lower nasal cartilages (Figure 11c,d) showed a dense structure of dilatator naris muscles attached to the perichondrium without young chondrocytes, while the upper regions (Figure 11e,f) displayed a thinner looser structure (Figure 12a) with numerous elastic fibres attached to a perichondrium with numerous young chondrocytes. Toluidine Blue staining in the TEM semi-thin samples confirmed that the cells are organized into isolated lobules with large round each containing a single chondrocyte (Figure 13) as previously observed [77].

These observations are in accordance with the mechanical dynamical analyses namely the tensile and compression results that confirm the different structural organization of collagen in the NS (Section 3.3). However, further studies are required in order to understand in detail the relation of these constituents in the different areas.

## 4. Conclusions

Regarding thermal features assessed by DSC and TGA, a lot of variability was found for NS, ULC, and LLC. However, the variability appears to be intrinsic to own tissues. Concerning the DSC analysis, it must be highlighted that a consistent peak occurred for all the samples between 100 °C and 130 °C due to water evaporation. In general, a higher temperature peak was detected in subcutaneous tissues comparatively to cartilages. This difference might be attributed to the higher water-binding capacity of subcutaneous tissue that results in higher difficulty for water to move out. Moreover, the consistent narrow peaks of the subcutaneous tissues may suggest different mechanisms of water-binding, and consequently, distinct corresponding viscoelastic behaviours. In TGA, the degradation curves of cartilage, composite, and subcutaneous tissues from NS, ULC, and LLC structures occurred in three well-defined stages. The first attributed to water loss showed a main average peak at 37 °C. The second related to tightly bonded water was between 163.7–196.3 °C. Finally, the third attributed to protein degradation was in the range of 279.3–303.1 °C. The dry residues variably changed from 8.2 wt % to 19.5 wt % (on average) up to 500 °C. An important conclusion from this thermal analysis is that the water content of nasal cartilages was estimated to be 42 wt % (on average), differently from other well-known hyaline cartilages.

Tensile and compressive DMA results of NS showed different behaviours. Under tensile loading, the viscoelastic performance of NS was ruled by a flow-independent mechanism produced by the solid matrix, which is strongly frequency-dependent (1–20 Hz), presenting a clear glass transition point at around 200 °C. Under unconfined compression, three relaxation peaks (at 120 °C, 180 °C, and 220 °C) were registered through tan∂ curve associated to a second-order phase transition within the amorphous region of collagen and melting of crystalline zones of collagen and proteoglycans matrices, respectively. However, since cartilage is also rather hydrated, these peaks can also be related to water mobility within the cartilage structure. In fact, under compressive loading, the material is ruled by a flow-dependent mechanism caused by the frictional forces of the interstitial fluid that flows throughout the matrix.

The histology and electron microscopy results are coherent with the physical constitution of the studied regions. Comparing the upper and lower nasal regions, an increase of thickness was observed in the different areas: perichondrium, subcutaneous tissue, and inferior epidermis in the inferior nasal region. Histological analysis in H&E (Hematoxylin and Eosin) and semi-thin images indicate a larger number of cells (chondrocytes), increased vascular regions, and dense nasal connective tissue.

A final issue regards the potentialities that can result from the outcomes of this study, in the sense that they can contribute to the development of artificial materials that mimic the mechanical behaviour of natural soft-tissues of the human nose.

## Figures and Tables

**Table 1:** Nasal septum (NS) specimen dimensions (mm)—average values used in dynamic tension and compression tests. Ten measurements were taken for each specimen along the sample width.

Sp. Name (NS)	Thickness		Width	Length
	Total (Cartilage + Subcutaneous)	Cartilage		
Tension (dog-bone)	1.03 ± 0.19	0.94 ± 0.11	1.81 ± 0.29	10.00 ± 0.10
Compression (flat disc)	1.30 ± 0.12	0.83 ± 0.05	7.00 ± 0.10 (diameter)	
